# Loss of heme oxygenase 2 causes reduced expression of genes in cardiac muscle development and contractility and leads to cardiomyopathy in mice

**DOI:** 10.1371/journal.pone.0292990

**Published:** 2023-10-16

**Authors:** Rengul Cetin-Atalay, Angelo Y. Meliton, Cevher Ozcan, Parker S. Woods, Kaitlyn A. Sun, Yun Fang, Robert B. Hamanaka, Gökhan M. Mutlu

**Affiliations:** 1 Department of Medicine, Section of Pulmonary and Critical Care Medicine, University of Chicago, Chicago, Illinois, United States of America; 2 Department of Medicine, Section of Cardiology, University of Chicago, Chicago, Illinois, United States of America; Second Xiangya Hospital, CHINA

## Abstract

Obstructive sleep apnea (OSA) is a common breathing disorder that affects a significant portion of the adult population. In addition to causing excessive daytime sleepiness and neurocognitive effects, OSA is an independent risk factor for cardiovascular disease; however, the underlying mechanisms are not completely understood. Using exposure to intermittent hypoxia (IH) to mimic OSA, we have recently reported that mice exposed to IH exhibit endothelial cell (EC) activation, which is an early process preceding the development of cardiovascular disease. Although widely used, IH models have several limitations such as the severity of hypoxia, which does not occur in most patients with OSA. Recent studies reported that mice with deletion of hemeoxygenase 2 (*Hmox2*^*-/-*^), which plays a key role in oxygen sensing in the carotid body, exhibit spontaneous apneas during sleep and elevated levels of catecholamines. Here, using RNA-sequencing we investigated the transcriptomic changes in aortic ECs and heart tissue to understand the changes that occur in *Hmox2*^*-/-*^ mice. In addition, we evaluated cardiac structure, function, and electrical properties by using echocardiogram and electrocardiogram in these mice. We found that *Hmox2*^*-/-*^ mice exhibited aortic EC activation. Transcriptomic analysis in aortic ECs showed differentially expressed genes enriched in blood coagulation, cell adhesion, cellular respiration and cardiac muscle development and contraction. Similarly, transcriptomic analysis in heart tissue showed a differentially expressed gene set enriched in mitochondrial translation, oxidative phosphorylation and cardiac muscle development. Analysis of transcriptomic data from aortic ECs and heart tissue showed loss of *Hmox2* gene might have common cellular network footprints on aortic endothelial cells and heart tissue. Echocardiographic evaluation showed that *Hmox2*^*-/-*^ mice develop progressive dilated cardiomyopathy and conduction abnormalities compared to *Hmox2*^*+/+*^ mice. In conclusion, we found that *Hmox2*^*-/-*^ mice, which spontaneously develop apneas exhibit EC activation and transcriptomic and functional changes consistent with heart failure.

## Introduction

Obstructive sleep apnea (OSA) is a common disorder with a prevalence of 24% in men and 9% in women (based on apnea-hypopnea index ≥5) [1]. Population based studies estimate that the prevalence of OSA with daytime sleepiness is 3–7% in men and 2–5% in women [2]. In addition to causing daytime sleepiness, OSA is an independent risk factor for hypertension, atherosclerotic cardiovascular disease and heart failure [1–15]. Despite improvements in our knowledge about the underlying molecular mechanisms by which OSA causes cardiovascular disease, our understanding is not complete.

Pathophysiologically, OSA is characterized by recurrent obstruction of upper airway leading to cessation of air flow resulting in intermittent hypoxia (IH), arousals, and activation of sympathetic nervous system [12, 13, 16, 17]. As a hallmark manifestation of OSA, IH plays an important role in the pathogenesis of OSA-related cardiovascular morbidity [18]. Therefore, exposure of rodents to IH is often used as a model of OSA to study the mechanisms by which OSA causes cardiovascular disease [19].

Using the IH exposure model in mice, we have recently studied the effect of IH on endothelial cell (EC) function [20]. EC activation is an early process in the pathogenesis of cardiovascular disease. Activated ECs express pro-inflammatory cytokines and cell adhesion molecules [21], which may then lead to leukocyte adhesion and activation, and platelet aggregation, all of which contribute to the development of cardiovascular disease [22–24]. We and others have previously linked EC activation with the blood flow dynamics in vasculature. ECs from aortic arch were activated while ECs from abdominal aorta were not [25–28]. Using IH exposure in mice as a model of OSA, we have found that IH causes EC activation [20]. However, this effect was not a direct effect of IH, but was indirectly mediated via the activation of sympathetic nervous system and release of catecholamines.

In addition to IH, recent studies suggest that mice with deletion of hemeoxygenase 2 (*Hmox2*^*-/-*^) may also be used as a model of OSA [29, 30]. HMOX2 is an enzyme that catalyzes the oxidative cleavage of heme leading to the generation of carbon monoxide [31]. In contrast to HMOX1, which is expressed at low levels in most tissues, HMOX2 is inducible and expressed in in the brain, testes, and gastrointestinal tract [32, 33]. HMOX2 has been shown to be important in oxygen sensing in carotid body [34] as *Hmox2*^*-/-*^ mice showed a blunted hypoxic ventilatory response [34]. Peng and colleagues have reported that *Hmox2*^*-/-*^ mice develop spontaneous apneas during sleep [29]. Furthermore, in a more recent study, they also showed that these mice have increased systemic levels of catecholamines [30]. These studies suggested that *Hmox2*^*-/-*^ mice can be used as a model to mimic sleep apnea. However, how the cardiovascular system may be affected in the *Hmox2*^*-/-*^ model of sleep apnea has not been studied. Here, we studied whether *Hmox2*^*-/-*^ mice exhibit EC activation similar to the IH model of OSA. We also investigated the transcriptomic changes in aortic ECs and heart tissue as well as the functional changes in heart function in *Hmox2*^*-/-*^ mice.

## Materials and methods

### Animals

All experiments and procedures involving animals were approved by the Institutional Animal Care and Use Committee at the University of Chicago (Protocol number 72573). We used mice with deletion of Hmox2 (*Hmox2*^*-/-*^) and their wildtype littermate controls (*Hmox2*^*+/+*^). All animal experiments were performed according to the Animal Research: Reporting of In Vivo Experiments (ARRIVE) guidelines [35]. Mice were euthanized using the euthanasia solution (Euthasol (pentobarbital sodium and phenytoin sodium)) followed by exsanguination and removal of vital organs. The method of euthanasia is consistent with the recommendations of the American Veterinary Medical Association Guidelines for the Euthanasia of Animals. After confirming anesthesia following the administration of euthanasia solution, we performed thoracotomy and first collected blood from the right ventricle for catecholamine levels. We then cut the inferior vena cava and flushed heart and vessels with phosphate buffered saline to remove remaining blood in the vasculature. We then harvested the hearts and aortas and placed them in cold plate containing phosphate buffered saline and dissected under microscope [20, 25, 36]. Heart tissue was frozen in liquid nitrogen for RNA isolation. Total RNA from aortic ECs was isolated by gentle and slow flushing with TRI Reagent inside of the aortic lumen with a blunt end needle insertion.

### RNA isolation and sequencing

RNA isolation, sequencing, and analysis were done as we have previously described [37]. We isolated RNA from aortic ECs using TRI Reagent (Zymo Research, R2050-1-200) and Zymo Direct-zol RNA Miniprep Kit (Zymo Research, catalog number R2053) [20]. Heart tissue total RNA was isolated from fast-frozen whole heart. The tissue was pulverized under liquid nitrogen in a stainless-steel mortar and pestle and the frozen tissue powder quantitatively transferred to test tubes. Total RNA was isolated with RNeasy Plus Mini Kit (Qiagen, 74134). Total RNA from aortic ECs and heart tissues were submitted to the University of Chicago Genomics Core Facility for sequencing with the Illumina NovaSEQ6000 sequencer (100bp paired-end). Sequencing read (FASTQ) files were generated and assessed for per base sequence quality using FastQC. RNA-seq reads were pseudoaligned using Kallisto v.0.44.0 the at University of Chicago, CRI Gardner high performance computing cluster [38]. The Kallisto index was made with default parameters and the GENCODE (Mouse Release M32, GRCm39) and was run in quant mode with default parameters. Following pseudoalignment, we computed gene abundances using R package tximport v.1.18.0 [39]. Differential expression was calculated between the groups using R package edgeR [40]. edgeR performs read count filtering, normalization, estimating dispersion, and identification of differentially expressed genes. Differential gene expression was considered significant for genes with an FDR-adjusted p-value ≤ 0.05 and fold change (FC) > 2. All volcano plots were drawn using ggplot2 R package. All heatmaps were generated with Pretty heatmaps R package pheatmap package from Z-score normalized expression values. An absolute fold change ≥ 2 and false discovery rate (FDR) adjusted p-value ≤ 0.05 were used to select and classify the significant DEGs. Pathway enrichment analyses were performed, and plots were created using R clusterProfiler package [41] or using Enrichr search engine web interface on Enrichr database [42]. All packages were run on RStudio (2021.09.0 Build 351) with R version 4.0.3 Source data for RNA-seq are accessible via GEO (GSE230725). https://www.ncbi.nlm.nih.gov/geo/query/acc.cgi?acc=GSE230725

Transcription factor (TF)—target gene interactions gene regulatory network enrichment analysis was done with R DoRothEA package using mouse regulons [43]. DoRothEA contains 470,711 TF-target interactions for 1396 TFs for 20,238 unique genes.

### Cardiac structure, function, and rhythm analysis

Transthoracic echocardiography (VisualSonics, Vevo 770–120, RMV707B probe, Toronto, Canada) was used to evaluate left ventricular (LV) systolic function and structure while mice were under light anesthesia (0.5–1% isoflurane). Two-dimensionally guided M-mode images of the LV were acquired in the parasternal long and short axes to measure LV cavity dimensions, anterior and posterior wall thicknesses, and fractional shortening. LV ejection fraction was calculated based on these measurements. Electrocardiogram (ECG) (PowerLab using LabChart software, AD Instruments) was performed to determine heart rate, rhythm and conduction intervals in mice. Electrodes were placed subcutaneously in the limbs for lead II position for continuous ECG monitoring up to 5 minutes in each session. RR interval, PR interval, QRS duration, and QT interval were recorded and analyzed from limb leads in lead II. AF, atrioventricular block (AVB), premature beats, pauses, supraventricular, and ventricular arrhythmias were documented on ECG recordings.

### Statistics

The data were analyzed in Prism 9 (GraphPad Software Inc., La Jolla, CA). All data are shown as mean ± standard deviation. Significance was determined by unpaired, two-tailed Student’s t-test (for comparisons between two samples) or by one-way ANOVA using Bonferroni’s correction for multiple comparisons. Statistical significance was defined as *p<0.05, **p<0.005, ***p<0.001, ****p<0.0001.

## Results

### *Hmox2*^*-/-*^ mice exhibit EC activation in aorta

Increasing evidence suggest that Hmox2 plays an important role in oxygen sensing [31, 44]. Furthermore, recent studies by Peng and colleagues have reported that *Hmox2*^*-/-*^ mice exhibit spontaneous apneas during sleep [29, 30]. They also showed that these mice have increased systemic levels of catecholamines [30]. These results suggested the suitability of *Hmox2*^*-/-*^ as a model of sleep apnea; however, the cardiovascular changes in *Hmox2*^*-/-*^ are not completely understood.

**Fig 1:**
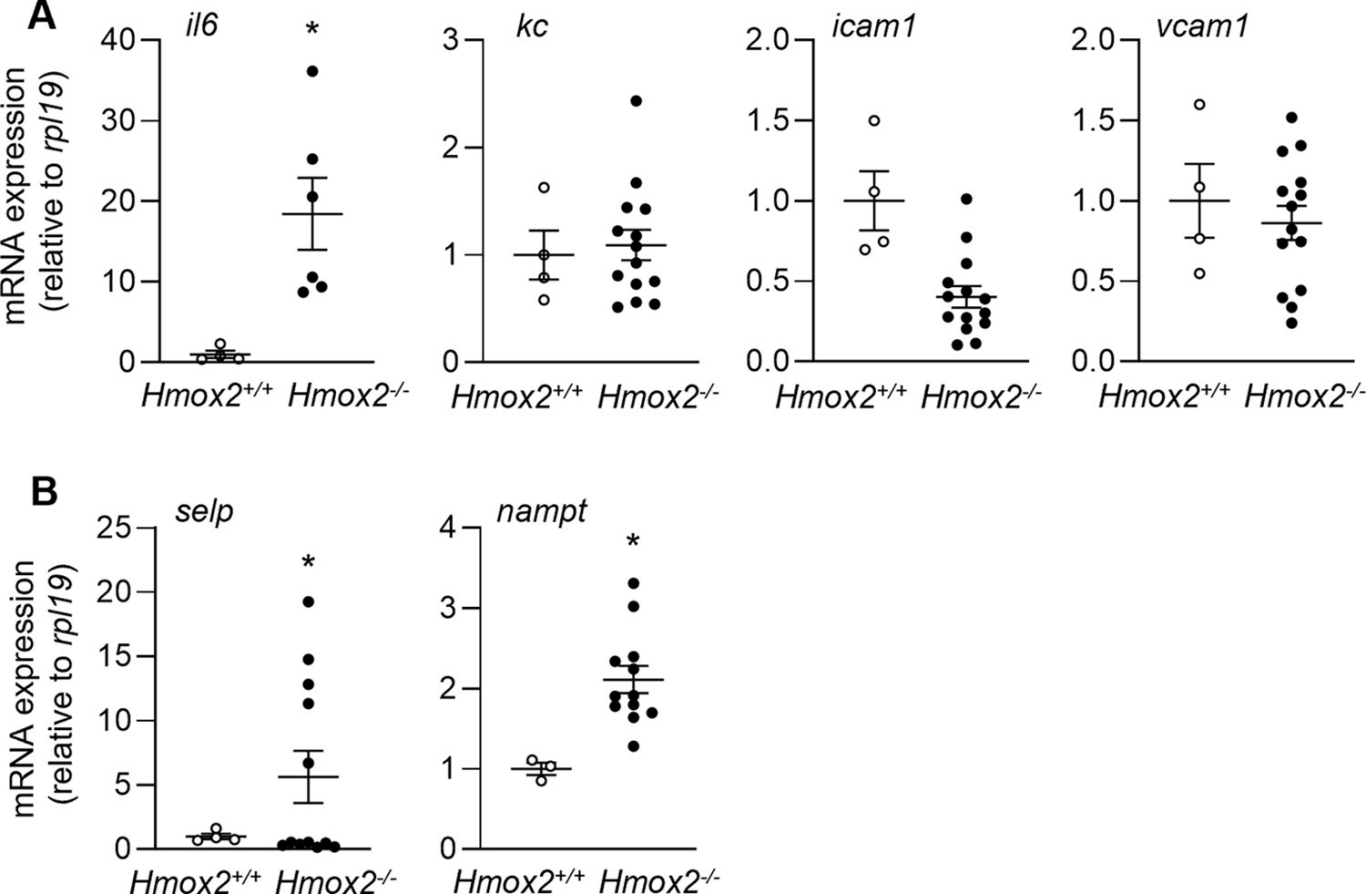
Loss of *Hmox2* is associated with endothelial cell activation. We isolated aortic endothelial cells from *Hmox2*^*-/-*^ and littermate control *Hmox2*^*+/+*^ mice and performed qPCR to measure expression of (A) cytokines and adhesion molecules (*il6*, *kc*, *icam1*, *vcam1*) and (B) other EC activation markers (*selp*, *nampt*) (n = 5/strain). *p<0.05.

We have recently reported that exposure to IH as a model of sleep apnea in mice is associated with aortic endothelial cell (EC) activation [20]. To determine whether *Hmox2* deficiency is associated with EC activation, we isolated aortic ECs from both *Hmox2*^*+/+*^ and *Hmox2*^*-/-*^ mice to measure expression of genes associated with EC activation. As previously reported [29, 30], compared to control *Hmox2*^*+/+*^ mice, *Hmox2*^*-/-*^ mice had spontaneous apneas and increased systemic levels of catecholamines (S1 Fig and S1 Text). We also confirmed the loss of *Hmox2* gene in aortic ECs isolated from *Hmox2*^*-/-*^ mice (S2 Fig and S1 Text). We found that compared to ECs from *Hmox2*^*+/+*^ mice, ECs from *Hmox2*^*-/-*^ mice exhibited increased expression of *il6*, but there was no increase in the expression of *Kc*, *Icam1* or *Vcam1* as we have previously reported in ECs isolated from wild-type mice exposed to IH [20] (Fig 1A). We also evaluated the expression of EC activation-associated genes *selp*, and *nampt*. *Selp* encodes P-selectin, which mediates the rolling of leukocytes on the surface of the endothelium and initiates their attachment to ECs [45]. Nicotinamide phosphoribosyltransferase (Nampt) is the rate-limiting enzyme of nicotinamide adenine dinucleotide salvage biosynthesis. *Nampt* has been shown to be associated with EC activation/dysfunction, vascular inflammation and progression of atherosclerosis [46, 47]. Consistent with EC activation in sleep apnea, we found that ECs from *Hmox2*^*-/-*^ mice showed increased expression of *selp* and *nampt* (Fig 1B). Collectively, these results suggested that aortic ECs from *Hmox2*^*-/-*^ mice exhibit gene expression changes consistent with EC activation.

### Loss of *Hmox2* is associated with DEGs enriched in blood coagulation, cell adhesion, cellular respiration, and cardiac muscle development and contraction in endothelial cells

**Fig 2:**
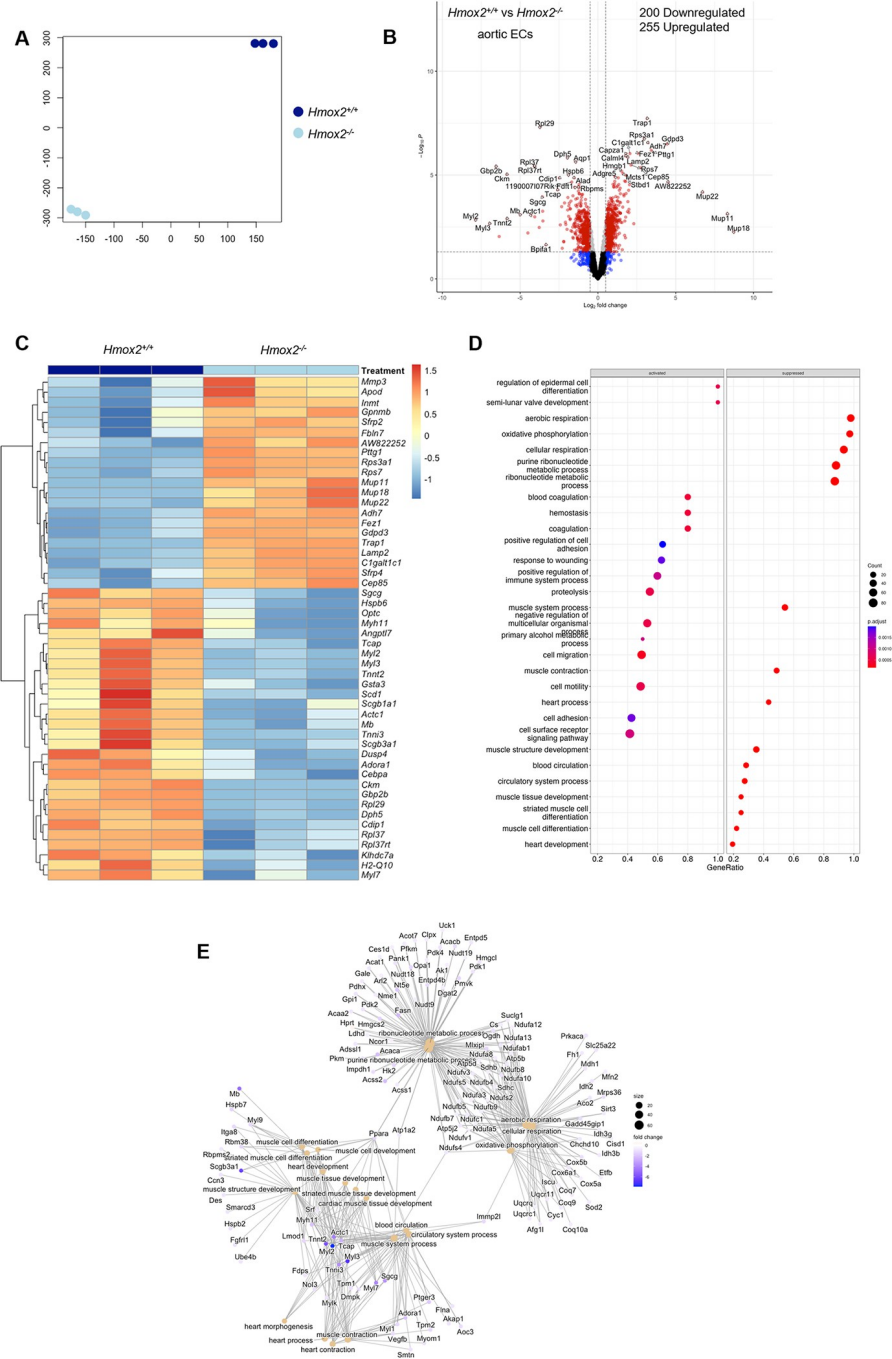
Transcriptional changes in aortic endothelial cells in *Hmox2*^*-/-*^ and *Hmox2*^*+/+*^ mice. RNA-sequencing was performed on aortic ECs collected from *Hmox2*^*-/-*^ and *Hmox2*^*+/+*^ mice (n = 3/strain). **(A)** Similarity level of high dimensional data of differentially expressed genes (DEGs) in aortic ECs from *Hmox2*^*-/-*^ and *Hmox2*^*+/+*^ mice visualized by t-sne plot of Log_2_ fold (LogFC) changes. **(B)** Volcano plot of DEGs (6151) and top significantly differentially regulated (255 up and 200 down) genes in *Hmox2*^*-/-*^. **(C)** Heat map of the top 50 significantly DEGs in aortic ECs. **(D)** Significantly activated and suppressed enriched gene sets of Gene ontology biological processes (GO_BP) terms enriched based on the significantly activated and suppressed genes in heart tissue. **(E)** Gene network associated with the identified biological processes in heart tissue. Gene set enrichment analysis (GSEA) parameters for significance were set to absolute fold change ≥2 and FDR adjusted p-value≤0.05 between *Hmox2*^*+/+*^ and *Hmox2*^*-/-*^. Source data for RNA-seq are accessible via GEO (GSE230725). https://www.ncbi.nlm.nih.gov/geo/query/acc.cgi?acc=GSE230725.

To better understand the effect of sleep apnea on ECs beyond their activation in the *Hmox2*^*-/-*^ model, we performed RNA-sequencing in ECs isolated from *Hmox2*^*-/-*^ and *Hmox2*^*+/+*^ mice. The t-sne plot showed good separation between samples from *Hmox2*^*-/-*^ and *Hmox2*^*+/+*^ mice based on differentially expressed genes (DEGs) (Fig 2A). Compared to ECs from *Hmox2*^*+/+*^ mice, the expression of 255 genes was upregulated, and 200 genes were downregulated in ECs from *Hmox2*^*-/-*^ mice (Fig 2B). Fig 2C shows the top 50 differentially expressed genes in ECs between *Hmox2*^*-/-*^ and *Hmox2*^*+/+*^ mice. Gene ontology biological processes (GO_BP) enrichment analysis showed that blood coagulation, hemostasis, positive regulation of cytokine production, activation of immune response and positive regulation of cell adhesion were activated in ECs from *Hmox2*^*-/-*^ mice (Fig 2D). In contrast, oxidative phosphorylation and cellular respiration were processes that were downregulated in ECs from *Hmox2*^*-/-*^ mice. Interestingly, muscle contraction and heart contraction were also processes that were downregulated. Similar processes and pathways were identified using the gene set enrichment analysis (GSEA) of DEGs in ECs from *Hmox2*^*-/-*^ mice including cellular respiration, oxidative phosphorylation, blood circulation, cardiac muscle cell development and muscle contraction, and ribonucleotide metabolic processes. Additionally, enrichment analysis of DEGs across GO, KEGG and MGI_phenotype multiple datasets by Enrichr-KG tool also significantly identified cardiomyopathy and oxidative phosphorylation KEGG pathways and abnormal glucose homeostasis MGI-Mouse phenotype along with the above stated GO terms (S3 Fig). These results suggested that ECs from *Hmox2*^*-/-*^ mice, a model of sleep apnea, have a significant number of induced DEGs enriched in coagulation and cell adhesion, further supporting EC activation, and repressed DEGs enriched in cellular respiration and, oxidative phosphorylation. Surprisingly, ECs from *Hmox2*^*-/-*^ mice have a significant number of DEGs enriched in cardiac muscle development and contraction.

### Loss of *Hmox2* is associated with DEGs enriched in mitochondrial translation, oxidative phosphorylation and cardiac muscle development in heart tissue

**Fig 3:**
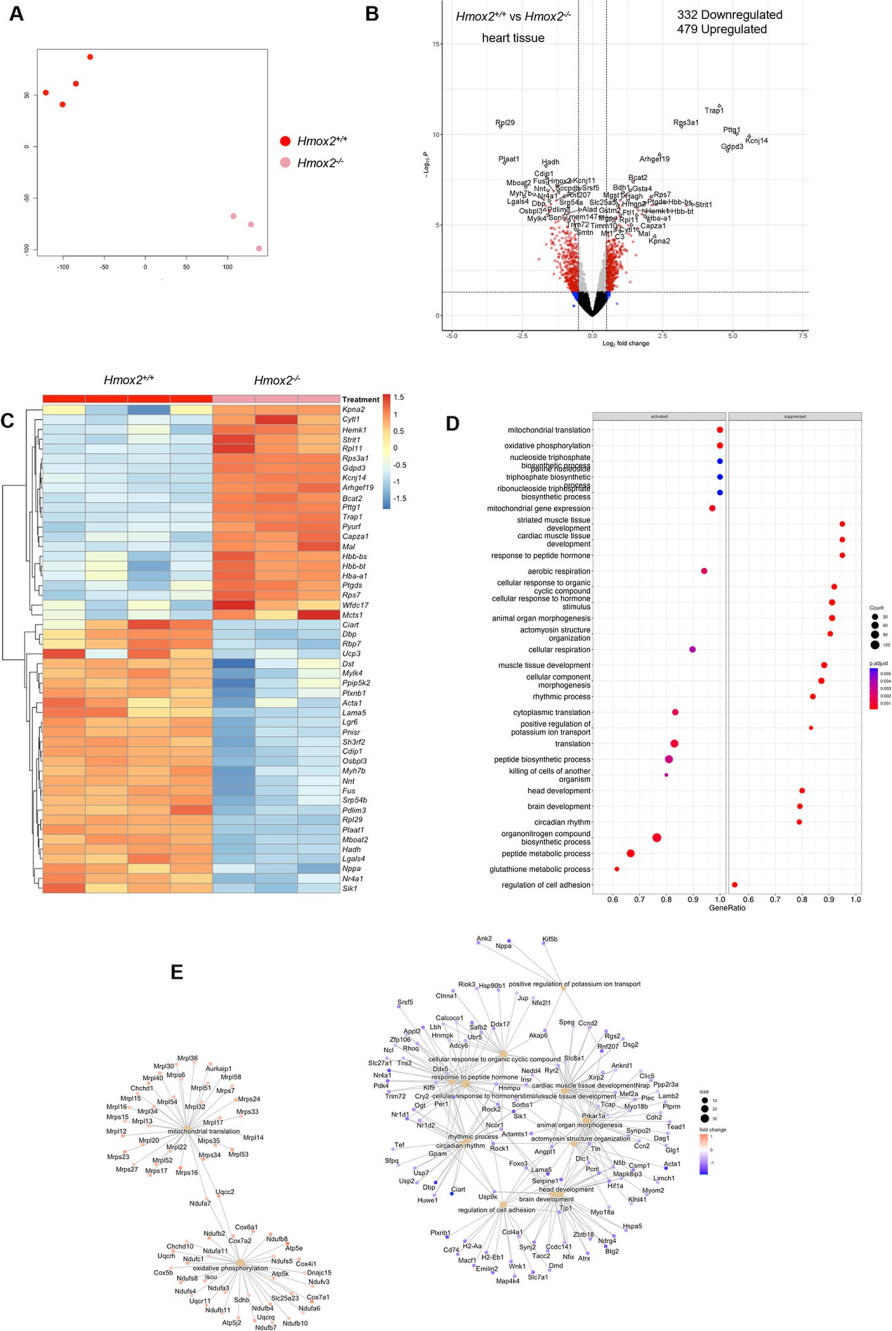
Transcriptional changes in heart tissue from *Hmox2*^*-/-*^ and *Hmox2*^*+/+*^ mice. RNA-sequencing was performed on heart tissues that were collected from *Hmox2*^*-/-*^ and *Hmox2*^*+/+*^ mice (n = 3/strain). **(A)** Similarity level of high dimensional data of DEGs in heart tissues from *Hmox2*^*-/-*^ and *Hmox2*^*+/+*^ mice visualized by t-sne plot of Log_2_ fold (LogFC) changes. **(B)** Volcano plot of DEGs (4422) and top significantly differentially regulated (479 up and 332 down) genes in *Hmox2*^*-/-*^. **(C)** Heat map of top 50 significant DEGs in heart tissue. **(D)** Gene ontology biological processes (GO_BP) terms enriched based on the significantly activated and suppressed genes in heart tissue. **(E)** Gene network associated with the identified biological processes in heart tissue. Gene set enrichment analysis (GSEA) parameters for significance were set to absolute fold change ≥2 and FDR adjusted p-value≤0.05 between *Hmox2*^*+/+*^ and *Hmox2*^*-/-*^. Source data for RNA-seq are accessible via GEO (GSE230725). https://www.ncbi.nlm.nih.gov/geo/query/acc.cgi?acc=GSE230725.

Surprisingly, the transcriptomic analysis of ECs showed reduced expression of genes in cardiac muscle development and contraction in *Hmox2*^*-/-*^ mice. Consistent with our data, a recent study in which the investigators performed single cell RNA-sequencing on mouse aorta reported the presence of a cluster of aortic ECs which express high levels of troponin and other myocyte markers [48]. These findings led us to perform RNA-sequencing in heart tissue from *Hmox2*^*-/-*^ and *Hmox2*^*+/+*^ mice. We confirmed the loss of *Hmox2* gene in heart tissue isolated from *Hmox2*^*-/-*^ mice (S2 Fig and S1 Text). The t-sne plot showed good separation between samples from *Hmox2*^*-/-*^ and *Hmox2*^*+/+*^ mice based on differentially expressed genes (DEGs) (Fig 3A). Compared to heart tissue from *Hmox2*^*+/+*^ mice, the expression of 479 genes was upregulated, and 332 genes were downregulated in heart tissue from *Hmox2*^*-/-*^ mice (Fig 3B). Fig 3C shows the top 50 DEGs in heart tissue between *Hmox2*^*-/-*^ and *Hmox2*^*+/+*^ mice. GO_BP enrichment analysis showed that mitochondrial translation, mitochondrial gene expression, aerobic respiration as well as nucleoside triphosphate biosynthetic process were activated in heart tissue from *Hmox2*^*-/-*^ mice (Fig 3D). Similar to ECs, cardiac muscle development was one of the processes downregulated in heart tissue from *Hmox2*^*-/-*^ mice (Fig 3D). GSEA of DEGs showed again mitochondrial translation, oxidative phosphorylation, and cardiac muscle development as pathways enriched in heart tissues from *Hmox2*^*-/-*^ mice. MGI-Mouse Phenotype terms of decreased skeletal muscle fiber diameter, abnormal muscle physiology and decreased cardiac muscle contractility were notably associated with the loss of *Hmox2* by the enrichment analysis with Enrichr-KG tool (S4 Fig), These results suggested that heart from *Hmox2*^*-/-*^ mice have a significant number of DEGs enriched in mitochondrial translation, oxidative phosphorylation and cardiac muscle development, which are similar to those we found in ECs.

### Shared pathway footprints and transcriptional regulation of gene expression in aortic ECs and heart tissue in *Hmox2*^-/-^ mice

**Fig 4:**
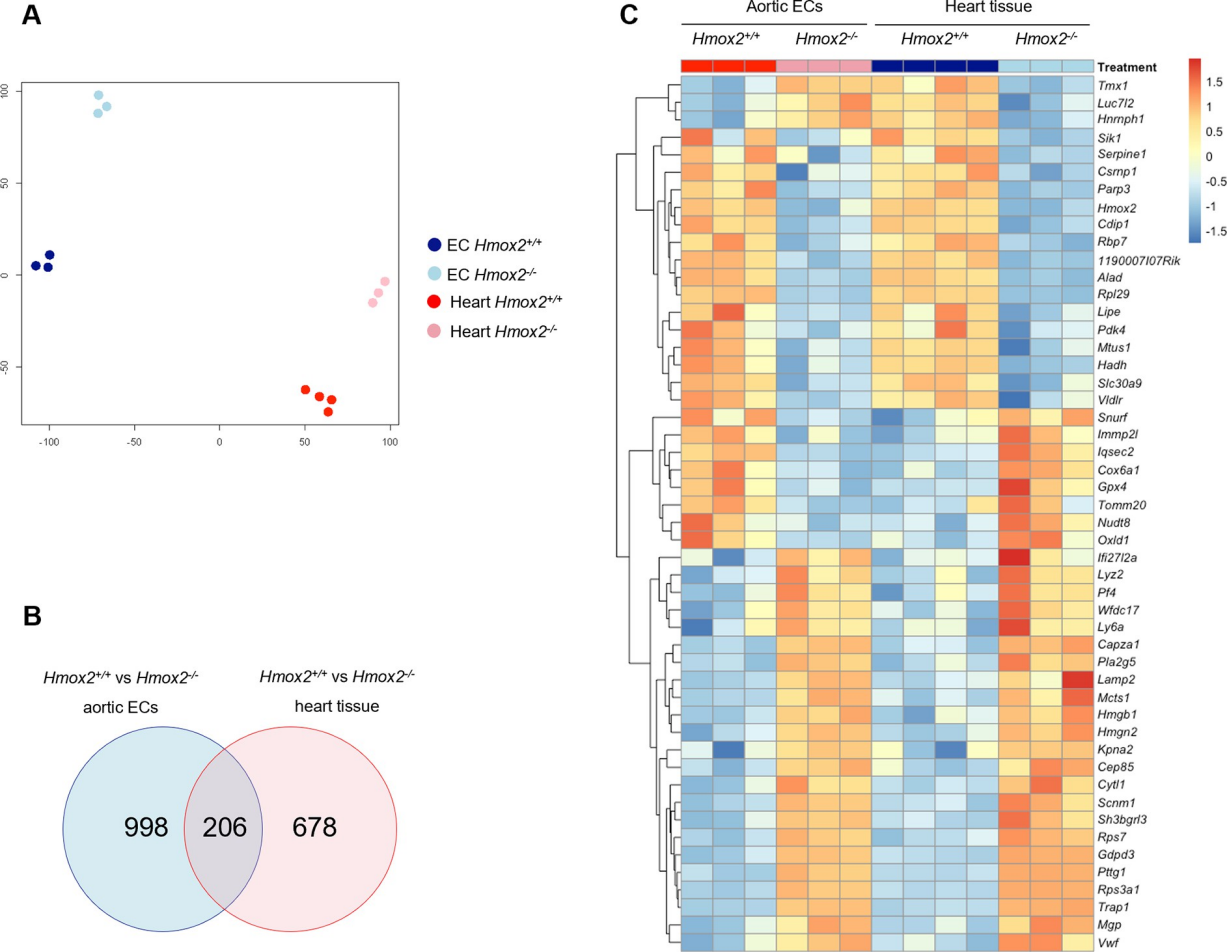
Combined gene expression analysis of aortic ECs and heart tissue from *Hmox2*^*-/-*^ and *Hmox2*^*+/+*^ mice. Read counts both from aortic ECs and heart tissue from *Hmox2*^*+/+*^ and *Hmox2*^*-/-*^ mice were analyzed in combination. **(A)** Similarity level of high dimensional data of differentially expressed genes (DEGs) in aortic ECs from *Hmox2*^*-/-*^ and *Hmox2*^*+/+*^ mice and heart tissues from *Hmox2*^*-/-*^ and *Hmox2*^*+/+*^ mice visualized by t-sne plot of Log_2_ fold (LogFC) changes. **(B)** Venn diagram of significant DEGs (logFC > = 0.5 p-value<0.05) in aortic ECs and heart tissue from *Hmox2*^*+/+*^ vs. *Hmox2*^*-/-*^ mice. **(C)** Heat map of top 50 genes from shared 206 DEGs between aortic ECs and heart tissue.

Transcriptome profiles from aortic ECs and heart tissue from *Hmox2*^*-/-*^ mice in comparison with their wild-type control littermates were very similar in terms of their GO_BP enrichments (Figs 2D and 3D). In addition, there were common genes in top 50 DEG sets between ECs and heart (Figs 2C and 3C). These findings suggested that these two RNA expression data from two different sources of the cardiovascular system might share common cellular network footprints in *Hmox2*^*-/-*^ mice. Therefore, we analyzed aortic ECs and heart tissue RNA expression profiles in combination. t-SNE analysis of RNAseq read counts from ECs and heart tissue from *Hmox2*^*+/+*^ and *Hmox2*^*-/-*^ mice showed that gene expression profiles were linearly separable for all 4 groups (Fig 4A). 206 DEGs were common in ECs and heart tissue when *Hmox2*^*-/-*^ compared to their *Hmox2*^*+/+*^ littermates (Fig 4B). The majority of the top 50 common DEGs had parallel expression regulation (Fig 4C). These findings suggest that deletion of *Hmox2* might have common cellular network footprints on aortic ECs and heart tissue.

**Fig 5:**
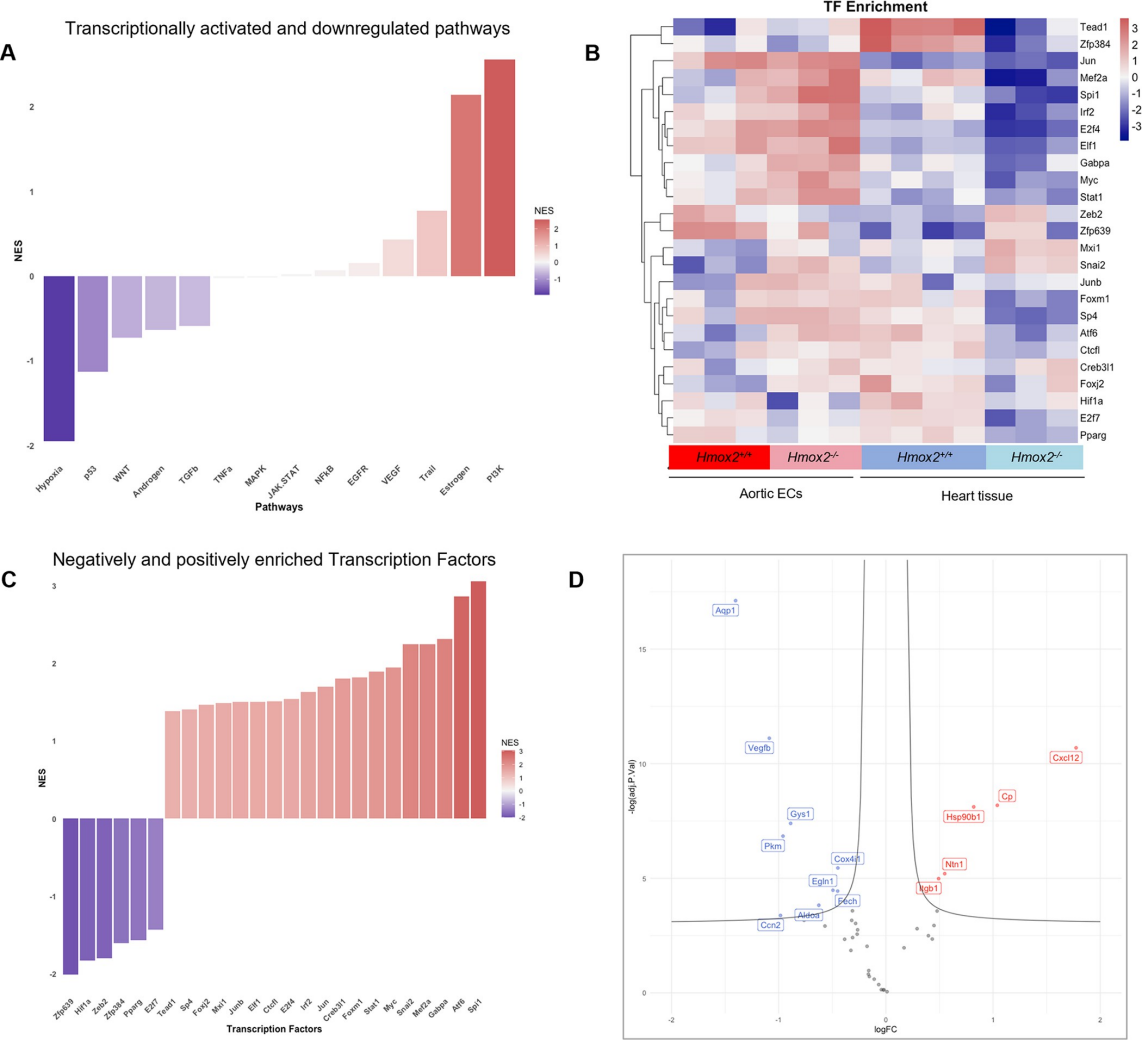
Shared gene alteration dependent gene regulatory network analysis of aortic ECs and heart tissue from *Hmox2*^*-/-*^ and *Hmox2*^*+/+*^ mice. **(A)** Significantly upregulated and downregulated PROGENy pathways. The color legend indicates the degree of enrichment. Normalized enrichment score: NES **(B)** Transcription factor (TF)-target gene interactions computed with DoRothEA R tool shown as TF enrichment. **(C)** Negatively and positively enriched TFs. The color legend indicates TF activity. **(D)** Volcano plots of *Hif1a* regulated targets for both aortic ECs and heart tissue in *Hmox2*^*-/-*^ mice.

We used the functional genomics tool PROGENy to analyze the transcriptional regulation of signaling pathways. PROGENy prioritizes the most responsive genes upon corresponding pathway perturbation as “footprint gene sets” rather than the number of genes involved in the pathway [49, 50]. While PI3K, Estrogen, Trail, VEGF and EGFR, cellular survival pathway footprints were activated, Hypoxia, p53, WINT cellular stress response pathway footprints were downregulated with the deletion of *Hmox2* (Fig 5A). These findings obtained from mice on room air and not under oxygen-related stress support a molecular sensory function of *Hmox2* and a role for this gene in sensing environmental stress.

Transcription factor (TF) activities of *Hmox2*^*-/-*^ mice were investigated to support cellular network footprints. Transcription factor (TF) activities were analyzed with DoRothEA TF-target interactions (regulons) tool and TF activities were inferred in parallel with pathway footprints (Fig 5B). Consistent with downregulation of hypoxia-associated pathways, transcription factor activity of hypoxia response gene *Hif1a* was significantly reduced in *Hmox2*^*-/-*^ tissues (Fig 5C). *Hif1a* target genes were significantly downregulated while TFs target genes involved in cell survival were upregulated when both *Hmox2*^*-/-*^ mice ECs and heart tissue transcriptome profiles analyzed in combination (Fig 5D).

### *Hmox2*^*-/-*^ mice develop progressive cardiomyopathy

**Fig 6:**
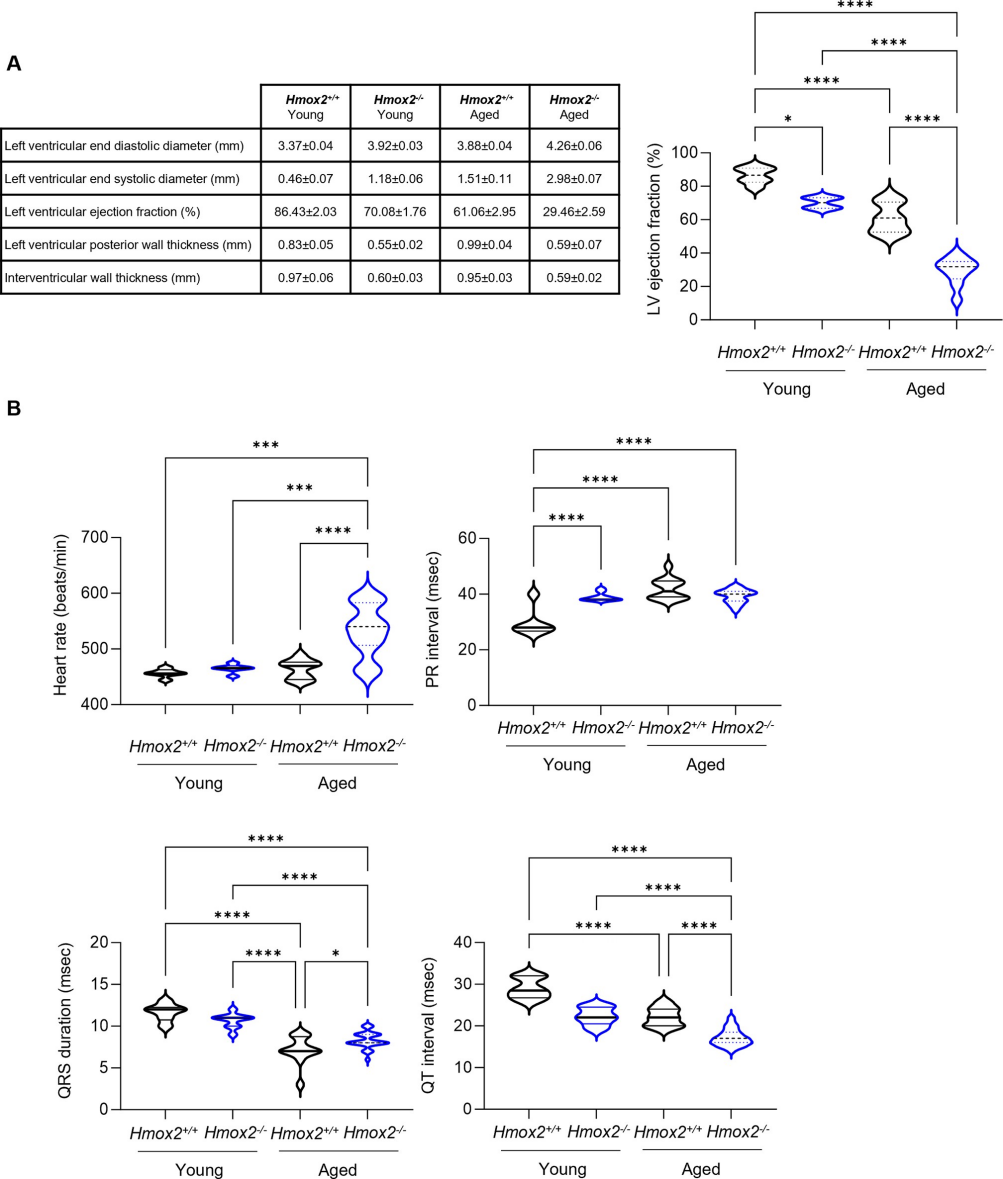
*Hmox2*^*-/-*^ mice develop dilated cardiomyopathy and conduction delay. We performed echocardiography on young (6–8 weeks old) and aged (6-months old) *Hmox2*^*-/-*^ and *Hmox2*^*+/+*^ mice. **(A)** Table shows LV end-diastolic, and systolic diameter, LV ejection, posterior wall and interventricular thickness and graph for LV ejection fraction (n = 3/group, *p<0.05). **(B)** We also performed electrocardiography on these mice. Heart rate, PR interval, QRS duration and QT interval are shown.

Since heart tissue from *Hmox2*^*-/-*^ mice showed decreased expression of genes involved in cardiac muscle development, we evaluated cardiac structure and function of these mice by using echocardiographic images. Left ventricle (LV) chamber size, wall thickness and systolic function were measured. Young (6–8 weeks old) or aged (6 months old) *Hmox2*^*-/-*^ mice developed significant LV dilation as shown by increased LV end diastolic diameter and LV end systolic diameter compared to *Hmox2*^*+/+*^ heart (n = 3 in each group, p<0.05 for all groups) (Fig 6A). This was associated with LV systolic dysfunction. As a marker of cardiac contractility, LV ejection fraction was significantly reduced in hearts of young (70.08±1.76%) and aged (29.46±2.59%) *Hmox2*^*-/-*^ mice versus hearts in *Hmox2*^*+/+*^ mice (86.43±2.03% in young and 61.06±2.95) (n = 3 in each group, p<0.05 for all groups). In parallel, we found that LV posterior wall thickness and interventricular septum thickness in young and aged *Hmox2*^*-/-*^ hearts were decreased. LV ejection fraction was significantly worsened through aging process of *Hmox2*^*-/-*^ mice. Also, LV dilation (systolic and diastolic) and wall thickness were deteriorated with aging of *Hmox2*^*-/-*^ mice. Thus, these morphological changes in *Hmox2*^*-/-*^ hearts were consistent with dilated cardiomyopathy and heart failure with reduced ejection fraction. We also studied cardiac electrical properties in all mice by analyzing ECG characteristics. Young *Hmox2*^*-/-*^ mice showed similar heart rate compared to *Hmox2*^*+/+*^ mice; however, ECG recordings showed higher heart rate in aged *Hmox2*^*-/-*^ mice. This increase in heart rate was likely due to reduced LV ejection fraction and cardiomyopathy. There was atrioventricular conduction delay in young and aged *Hmox2*^*-/-*^ mice as reflected by prolonged PR interval; however, QRS duration was comparable in *Hmox2*^*-/-*^ and *Hmox2*^*+/+*^. Hearts from young and aged *Hmox2*^*-/-*^ mice showed a repolarization abnormality with shorter QT interval compared to hearts from control mice. These results are consistent with the development of dilated cardiomyopathy in *Hmox2*^*-/-*^ mice and are also in agreement with the transcriptomic data showing DEGs enriched in cardiac muscle development.

## Discussion

OSA is a highly prevalent condition affecting up to 20% of the adult population in the US [1]. Recent estimates show that OSA is present in approximately 1 billion people in the world [51]. Clinically, OSA may present with neurocognitive changes including excessive daytime sleepiness. In addition, OSA is an independent risk factor for cardiovascular disease including hypertension, coronary artery disease and heart failure [1, 2, 7–15]. The pathophysiology of OSA includes repetitive upper airway collapse resulting in IH, intrathoracic pressure swings, arousals and activation of the sympathetic nervous system. Although the mechanisms of cardiovascular disease in OSA are not completely understood, both IH and increased catecholamines due to activation of the sympathetic nervous system have been implicated in the pathogenesis of cardiovascular disease. EC activation is an early process in the pathogenesis of atherosclerosis. We have recently reported that IH induces EC activation [20]; however, we found that IH-induced EC activation was not a direct effect of IH but required sympathetic nervous system activation and catecholamine release [20].

Exposure to IH is often used as a model to study the mechanisms of OSA. Although it is widely used, the IH model also has limitations [52]. IH does not reproduce all the physiological changes that occur in patients with OSA, such as increased respiratory efforts, intrathoracic pressure swings and hypercapnia. Importantly, IH models induce severe hypoxemia that does not occur in most patients with OSA. Peng and colleagues have recently described *Hmox2*^*-/-*^ mice as a spontaneous model of sleep apnea [29]. They also reported that *Hmox2*^*-/-*^ mice not only develop spontaneous apneas but also exhibit elevated levels of catecholamines [29, 30].

In this study, we investigated the transcriptional changes that occur in aortic ECs in *Hmox2*^*-/-*^ mice to better understand the impact of OSA and increased catecholamines on EC function. We found that aortic ECs from *Hmox2*^*-/-*^ mice exhibit hallmarks of activation. RNA-sequencing demonstrated that compared to aortic ECs from *Hmox2*^*+/+*^ mice, ECs from *Hmox2*^*-/-*^ mice had a significant number of DEGs enriched in blood coagulation, hemostasis, positive regulation of cytokine production, activation of immune response and cell adhesion, which were also consistent with EC activation and dysfunction and vascular inflammation. In addition, oxidative phosphorylation and cellular respiration were processes that were downregulated in ECs from *Hmox2*^*-/-*^ mice. These results are consistent with the published data showing EC activation with other models of OSA including IH [20, 53, 54]. They are also in agreement with Bellner et al. who showed that aortic ECs from *Hmox2*^*-/-*^ mice exhibit increased expression of pro-inflammatory cytokines and nuclear factor κB activation, which are consistent with EC activation [55].

Analysis of transcriptomic data also showed enrichment of DEGs in cardiac muscle development and contraction, which were downregulated in ECs from *Hmox2*^*-/-*^ mice. These surprising findings were consistent with a recently published data using single cell RNA-sequencing in mouse aortic ECs [48]. Lukowski and colleagues identified 4 different clusters of ECs, one of which was a cluster of aortic ECs which had high expression of cardiomyocyte genes such as troponin [48]. It is possible that these genes may be expressed in a group of ECs close to heart in the aortic arch. These findings led us to perform RNA-sequencing in heart tissue from *Hmox2*^*-/-*^ and *Hmox2*^*+/+*^ mice. Compared to control, heart tissue from *Hmox2*^*-/-*^ mice had DEGs enriched in mitochondrial gene expression and translation, and aerobic respiration. Similar to what we found in ECs, cardiac muscle development was one of the processes downregulated in heart tissue from *Hmox2*^*-/-*^ mice. Furthermore, MGI-Mouse Phenotype terms of decreased skeletal muscle fiber diameter, abnormal muscle physiology and decreased cardiac muscle contractility were associated with the loss of *Hmox2*. The gene expression changes that we observed in heart tissue from *Hmox2*^*-/-*^ mice are in agreement with the changes that occur in cardiomyocytes exposed to *IH in vitro* [56–59]. Exposure to IH causes cardiac inflammation and injury and decreases the viability of cardiomyocytes [56–59].

Given that genes that were involved in cardiomyocyte development and contractility were downregulated in *Hmox2*^*-/-*^ mice, we then evaluated whether *Hmox2*^*-/-*^ mice had any functional limitations. Echocardiographic evaluation showed structural and functional changes consistent with dilated cardiomyopathy, which progressed with aging. Collectively, we found that *Hmox2*^*-/-*^ mice, which spontaneously develop apneas and increased systemic level of catecholamines have EC activation, and cardiac dysfunction.

Integrative analysis of the transcriptomes from aortic ECs and heart tissue showed that they share common network footprints. Using functional genomics tools, we found that PI3K, Estrogen, Trail, VEGF and EGFR, cellular survival pathway footprints were activated, while hypoxia, p53, WINT cellular stress response pathway footprints were downregulated with the deletion of *Hmox2*. Consistent with the downregulation of the hypoxia pathway, analysis of transcription factors showed reduced activity of HIF1α in samples from *Hmox2*^*-/-*^ mice.

Our study had several limitations. First, we did not have another model of OSA such as IH exposure in wild-type mice as a control group limiting the translation of our findings in *Hmox2*^*-/-*^ mice to OSA. Since *Hmox2* is deleted in ECs and heart tissue, the effect of loss of *Hmox2* on ECs and heart tissue may not be solely due to increased apneas but may also be caused by the loss of Hmox2 gene in these cells/tissues. Further studies are warranted to determine whether the effects of IH are similar to those observed in *Hmox2*^*-/-*^ mice. Second, we also used bulk RNA-sequencing in heart tissue instead of single cell RNA-sequencing. Since the majority of the cells in heart tissue are cardiomyocytes, the bulk RNA-sequencing likely provided information of cardiomyocytes but the signal from other cells in the heart tissue including coronary ECs, macrophages, and fibroblasts were likely lost. Third, while we showed progression of dilated cardiomyopathy in aged mice, our sequencing data were obtained from young mice. Finally, we did not study the mechanisms underlying the cardiac changes we observed in *Hmox2*^*-/-*^ mice. HMOX2 has been implicated in the regulation of inflammation, redox sensing, oxidative stress and wound healing [33, 60–64]. Loss of Hmox2 leads to increased leukocyte infiltration and inflammatory cytokines in injury models [33, 60–63]. It is not completely understood how Hmox2 may regulate inflammation and oxidative stress; however, it may be due to the toxic effects of heme that could not be catabolized or buffered by Hmox2 [65].

In conclusion, our data in *Hmox2*^*-/-*^ mice, a spontaneous OSA model, showed expression of genes consistent with EC activation, downregulation of genes involved in cardiac muscle development and contractility, and developed progressive cardiomyopathy. While enrichment of DEGs in aerobic respiration suggested a change in mitochondrial function as a mechanism for the cardiac changes in *Hmox2*^*-/-*^ mice, further studies will be needed to better understand the mechanisms leading to heart failure in these mice.

## Supporting information

S1 Fig *Hmox2*^*-/-*^ mice exhibit spontaneous apneas and have increased systemic levels of catecholamines. We measured **(A)** apnea index using body plethysmography and **(B)** catecholamine levels in plasma in *Hmox2*^*+/+*^ and *Hmox2*^*-/-*^ mice (n = 5 for *Hmox2*^*+/+*^ and n = 10 for *Hmox2*^*-/-*^). *p<0.05. NE: norepinephrine, Epi: epinephrine.
(TIF)

S2 Fig Confirmation of loss of *Hmox2* expression in aortic ECs and heart tissue from *Hmox2*^*-/-*^ mice. We measured mRNA expression of *Hmox2* using qPCR in **(A)** aortic ECs and **(B)** heart tissue from *Hmox2*^*+/+*^ and *Hmox2*^*-/-*^ mice.
(TIF)

S3 Fig Enrichment analysis of DEGs from mouse aortic ECs. Enrichment analysis of significantly (LogFC ≥1 and p≤0.05) DEGs from RNA-sequencing data from aortic ECs isolated from *Hmox2*^*-/-*^ and *Hmox2*^*+/+*^ mice (n = 3/strain), across GO, KEGG and MGI-Mouse phenotype datasets by Enrichr-KG tool. Nodes are genes (green) and functional terms, edges connect genes to their enriched terms in the enrichment graph.
(TIF)

S4 Fig Enrichment analysis of DEGs from mouse heart tissue. Enrichment analysis of significantly (LogFC ≥1 and p≤0.05) DEGs from RNA-sequencing data from mouse heart tissue from *Hmox2*^*-/-*^ and *Hmox2*^*+/+*^ mice (n = 3/strain), across GO, KEGG and MGI-Mouse phenotype datasets by Enrichr-KG tool. Nodes are genes (green) and functional terms, edges connect genes to their enriched terms in the enrichment graph.
(TIF)

S1 Text Additional methods and results. We provide methods on the measurement of apnea index, plasma catecholamines and qPCR for *Hmox2*. We also show the results about the apnea index, plasma catecholamine levels and Hmox2 expression in aortic ECs and heart tissue from *Hmox2*^*-/-*^ mice.
(DOCX)
