# Supplementary figures and images for: Loss of heme oxygenase 2 causes reduced expression of genes in cardiac muscle development and contractility and leads to cardiomyopathy in mice
Source: PLoS One. 2023 Oct 16;18(10):e0292990. doi: 10.1371/journal.pone.0292990 (PMC10578579; doi:10.1371/journal.pone.0292990)

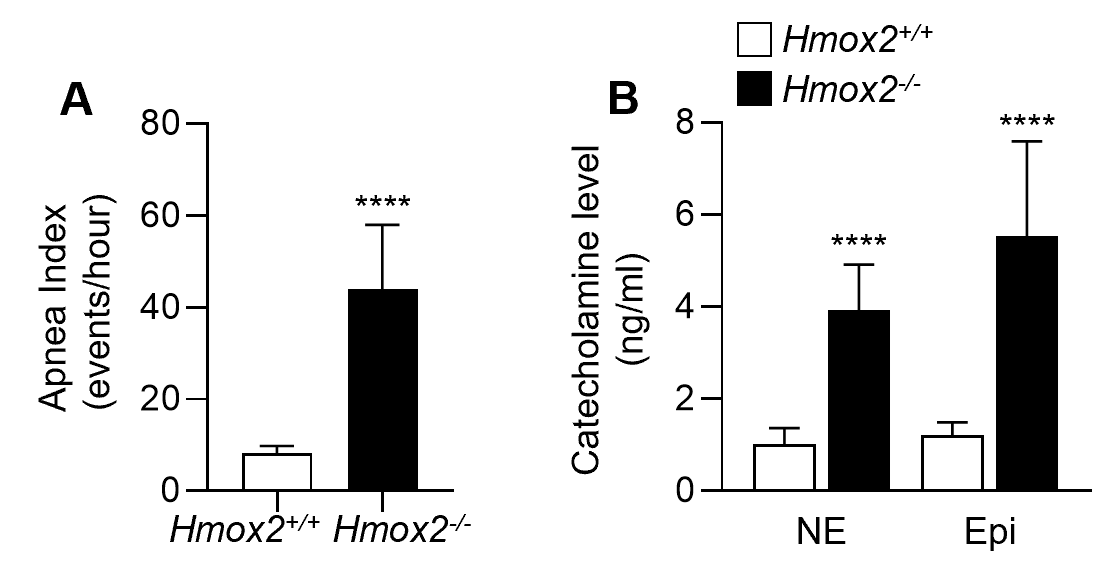

Supplement: S1 Fig — We measured (A) apnea index using body plethysmography and (B) catecholamine levels in plasma in Hmox2+/+ and Hmox2-/- mice (n = 5 for Hmox2+/+ and n = 10 for Hmox2-/-). *p<0.05. NE: norepinephrine, Epi: epinephrine. (TIF) [file pone.0292990.s001.tif]

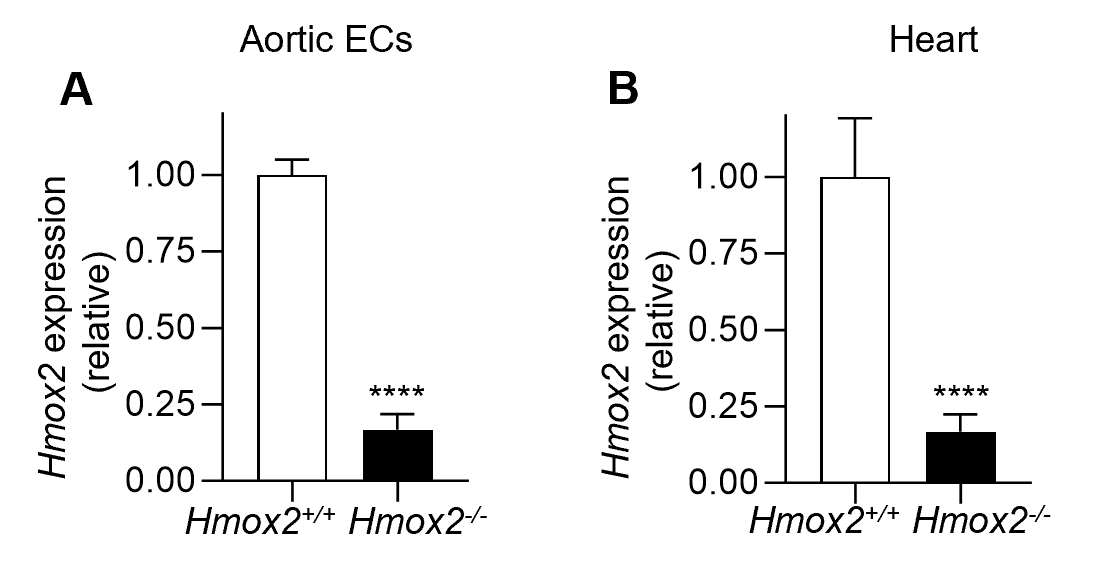

Supplement: S2 Fig — We measured mRNA expression of Hmox2 using qPCR in (A) aortic ECs and (B) heart tissue from Hmox2+/+ and Hmox2-/- mice. (TIF) [file pone.0292990.s002.tif]

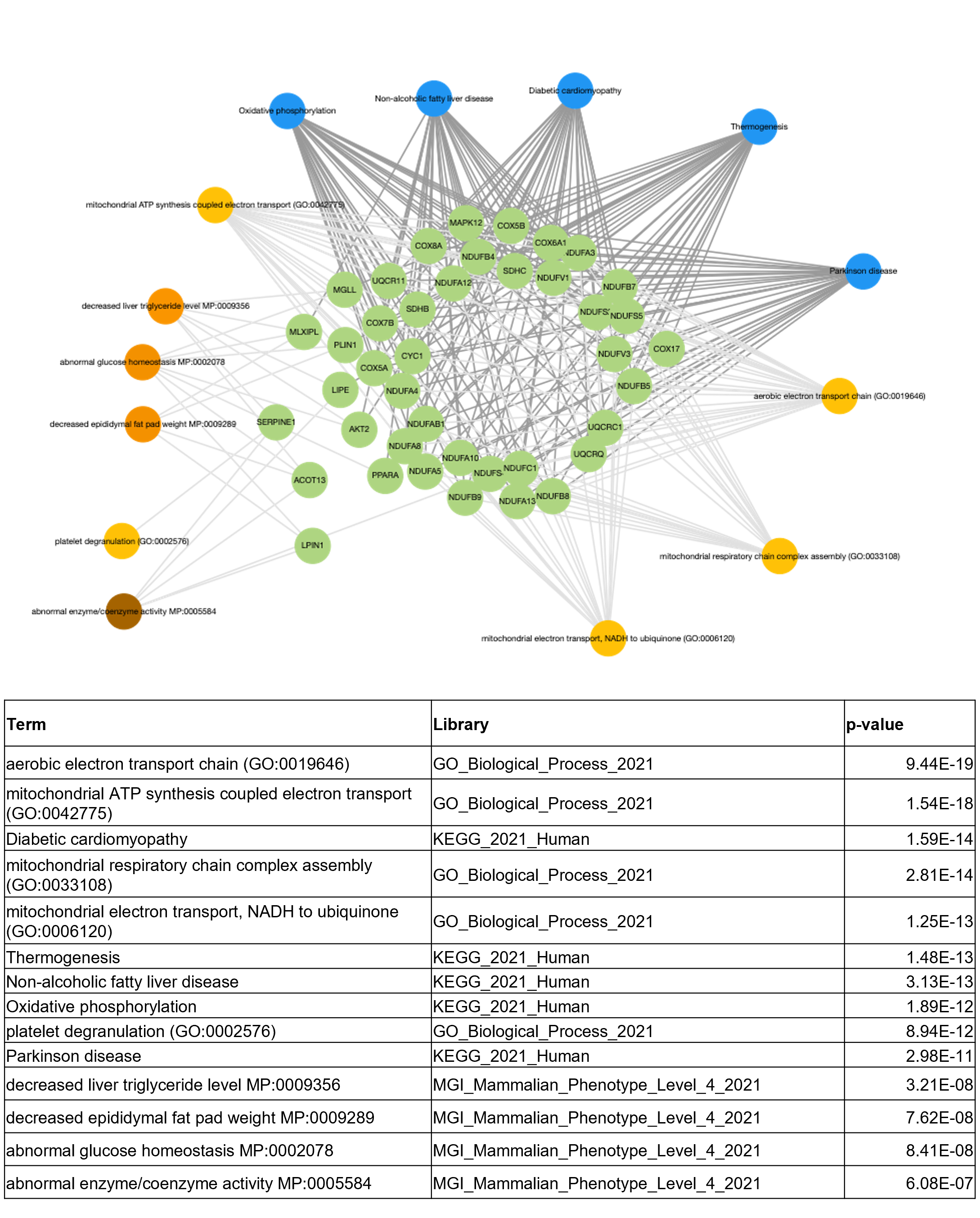

Supplement: S3 Fig — Enrichment analysis of significantly (LogFC ≥1 and p≤0.05) DEGs from RNA-sequencing data from aortic ECs isolated from Hmox2-/- and Hmox2+/+ mice (n = 3/strain), across GO, KEGG and MGI-Mouse phenotype datasets by Enrichr-KG tool. Nodes are genes (green) and functional terms, edges connect genes to their enriched terms in the enrichment graph. (TIF) [file pone.0292990.s003.tif]

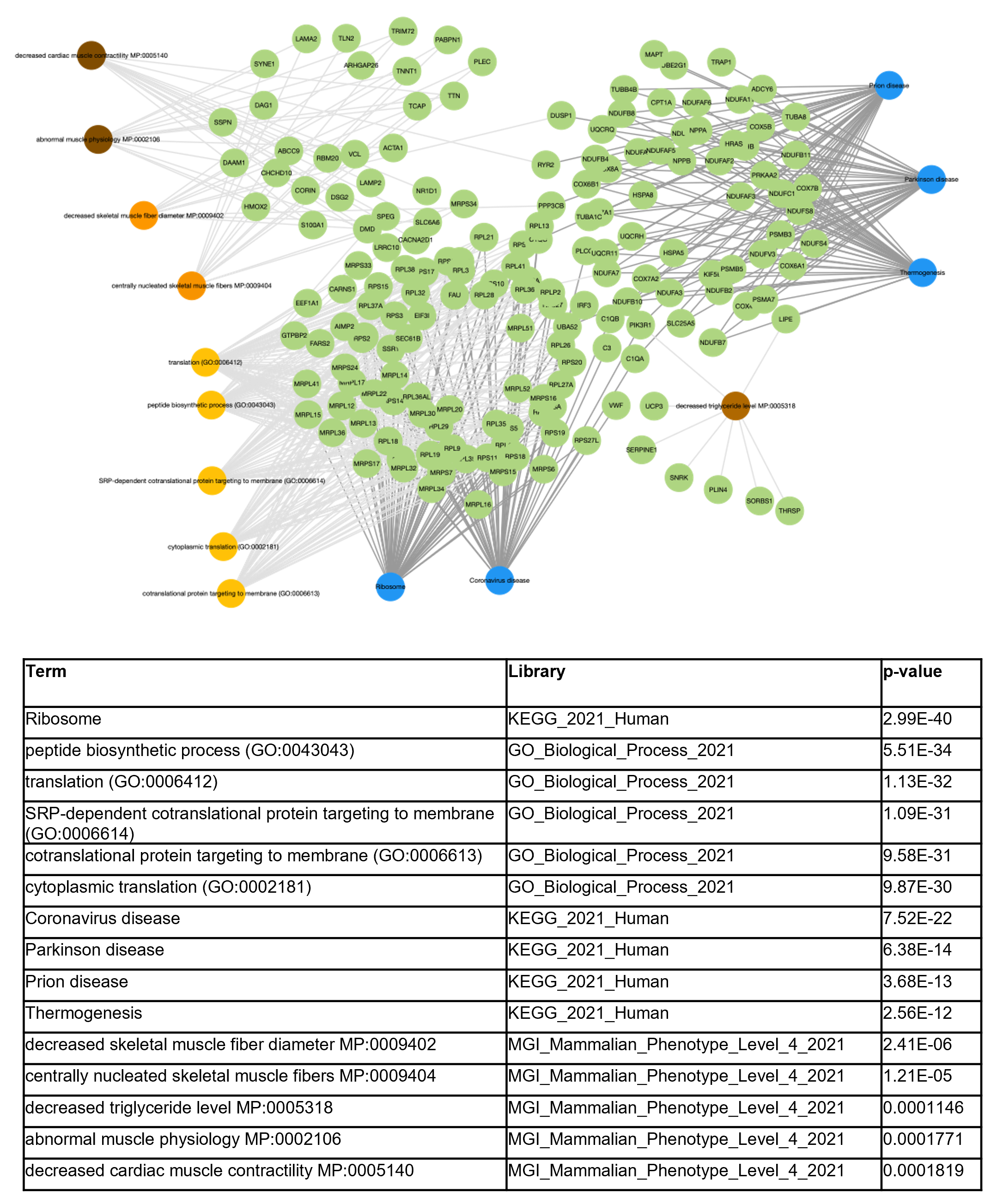

Supplement: S4 Fig — Enrichment analysis of significantly (LogFC ≥1 and p≤0.05) DEGs from RNA-sequencing data from mouse heart tissue from Hmox2-/- and Hmox2+/+ mice (n = 3/strain), across GO, KEGG and MGI-Mouse phenotype datasets by Enrichr-KG tool. Nodes are genes (green) and functional terms, edges connect genes to their enriched terms in the enrichment graph. (TIF) [file pone.0292990.s004.tif]
