# Supplementary material for: Loss of heme oxygenase 2 causes reduced expression of genes in cardiac muscle development and contractility and leads to cardiomyopathy in mice
Source: PLoS One. 2023 Oct 16;18(10):e0292990. doi: 10.1371/journal.pone.0292990 (PMC10578579; doi:10.1371/journal.pone.0292990)
Supplement: S1 Text — We provide methods on the measurement of apnea index, plasma catecholamines and qPCR for Hmox2. We also show the results about the apnea index, plasma catecholamine levels and Hmox2 expression in aortic ECs and heart tissue from Hmox2-/- mice. (DOCX) [file pone.0292990.s005.docx]

**Methods**

**Measurement of apnea index**

Apnea index (AI) was determined by recording breathing by whole body plethysmograph (SCIREQ, Montreal, QC, Canada) in unsedated *Hmox2^-/-^* mice and control mice breathing room air as previously described [30]. All measurements were made between 10:00 AM and 3:00 PM at an ambient temperature of 25°C ± 1°C. Apnea is defined as cessation of breathing longer than the duration of 2.5 normal breaths.

**Measurements of plasma catecholamines**

Blood samples (∼300 μL) were collected and placed in heparinized (30 U/mL of blood) and plasma was separated. Plasma Catecholamines was determined by high pressure liquid chromatography combined with electrochemical detection (HPLC-ECD) using dihydroxybenzylamine as an internal standard as described. The Catecholamines levels were normalized to 100 mL of plasma.

**Expression of *Hmox2* mRNA in aortic ECs and heart**

Loss of *Hmox2* expression was confirmed with quantitative mRNA expression by real-time quantitative PCR (qPCR) using iTaq Universal SYBR Green Supermix (catalog number 172-5121; Bio-Rad). *Rpl19* (Ribosomal protein L19) was used as a housekeeping gene, and gene expression was quantified using the ΔΔCt method. The mouse-specific primer sequences used for quantitative PCR are:

*Hmox2* (5’- TCGGAGGGGGTAGATGAGTC -3’, 5’- GCTTCCTTGGTCCCTTCCTT -3’),

*Rlp19* (5’-CCGACGAAAGGGTATGCTCA-3’, 5’-GACCTTCTTTTTCCCGCAGC-3’).

**Results**

**Mice lacking *Hmox2* exhibit apneas and increased systemic levels of catecholamines**

Increasing evidence suggest that Hmox2 plays an important role in oxygen sensing [31, 43]. Furthermore, recent studies by Peng and colleagues have reported that *Hmox2^-/-^* mice exhibit increased numbers of apneas during sleep [29, 30]. Furthermore, in a more recent study, they also showed that these mice have increased systemic levels of catecholamines [30]. We performed plethysmography to determine the presence of respiratory events during sleep in *Hmox2^-/-^* mice as previously described [30]. We confirmed that *Hmox2^-/-^* mice exhibit increased numbers of apneas with an apnea index of 43.99 per hour consistent with severe sleep disordered breathing (S1 Fig). We also measured plasma levels of catecholamines, which were elevated in *Hmox2^-/-^* mice compared to control mice *Hmox2^+/+^* mice (S1 Fig). These results confirmed that suitability of *Hmox2^-/-^* mice as a model of sleep apnea, which is associated not only with apnea or hypopneas but also with activation of sympathetic nervous system leading to increased systemic levels of catecholamines.

**Confirmation of loss of *Hmox2* expression aortic ECs and heart tissue from *Hmox2^-/-^* mice.** To confirm the loss of *Hmox2* expression, we isolated RNA from aortic ECs and heart tissue from *Hmox2^+/+^* and *Hmox2^-/-^* mice and performed qPCR. We found that *Hmox2* expression was significantly reduced in **(A)** aortic ECs and **(B)** heart tissue from *Hmox2^-/-^* mice.
